# Shape and Charge of Gold Nanomaterials Influence Survivorship, Oxidative Stress and Moulting of *Daphnia magna*

**DOI:** 10.3390/nano6120222

**Published:** 2016-11-25

**Authors:** Fatima Nasser, Adam Davis, Eugenia Valsami-Jones, Iseult Lynch

**Affiliations:** School of Geography Earth and Environmental Sciences, University of Birmingham, Edgbaston, Birmingham B15 2TT, UK; ac3443@coventry.ac.uk (A.D.); e.valsamijones@bham.ac.uk (E.V.-J.); i.lynch@bham.ac.uk (I.L.)

**Keywords:** nano-safety, *Daphnia magna*, surface charge, ecotoxicology, oxidative stress

## Abstract

Engineered nanomaterials (ENMs) are materials with at least one dimension between 1–100 nm. The small size of ENMs results in a large surface area to volume ratio, giving ENMs novel characteristics that are not traditionally exhibited by larger bulk materials. Coupled with large surface area is an enormous capacity for surface functionalization of ENMs, e.g., with different ligands or surface changes, leading to an almost infinite array of variability of ENMs. Here we explore the effects of various shaped (spheres, rods) and charged (negative, positive) gold ENMs on *Daphnia magna* (*D. magna*) in terms of survival, ENM uptake and production of reactive oxygen species (ROS), a key factor in oxidative stress responses. We also investigate the effects of gold ENMs binding to the carapace of *D. magna* and how this may induce moulting inhibition in addition to toxicity and stress. The findings suggest that ENM shape and surface charge play an important role in determining ENM uptake and toxicity.

## 1. Introduction

The incorporation of engineered nanomaterials (ENMs) into industrial products has significantly increased due to their enhanced reactivity characteristics, many of which are provided by an increased surface-area-to-volume ratio compared to larger micron or bulk material of the same chemical composition [1,2]. ENMs are being incorporated into various fields such as medicine [3] and environmental remediation [4]. Different shapes and morphologies of ENMs features provide further enhanced qualities, such as hollowness for use in drug delivery for the transport of materials [5]. There has been considerable progress towards the incorporation of spherical gold ENMs into medical applications, for example, by using gold ENMs to bind antibodies specifically targeted for cancer cells [6]. Gold nano-rods are also being tested for use in cancer therapy, and are found to have the highest absorption efficiency (~14), which refers to their heating capacity, compared to nano-spheres or silica-gold nano-shells with absorption efficiencies ranging between 3.5–4.02 [6]. Gold ENMs with a high aspect ratio (rods) are better photoabsorbers than spherical gold ENMs, and can convert the absorbed light to localized heath to photothermally destruct cancer cells [7]. Due to the increased use of various shaped gold ENMs in medical research, where the annual estimated consumption of nanoscale gold particles is 540 kg in the United Kingdom, which equals approximately 4.27 × 10^20^ ENMs assuming ENMs are 50 nm, the predicted environmental concentration (PEC) in surface waters is estimated at 468 pg/L [8] and therefore the deposition of gold ENMs into the environment and their subsequent interaction with organisms is a growing concern.

*Daphnia magna* (*D. magna*) occupy an important role in fresh water ecosystems as they ingest content present in the water column. It has been documented that 20 nm (0.04 nM ≈ 7.88 µg/L) spherical gold ENMs are taken up by *D. magna* reaching a steady-state concentration within the gut of *D. magna* in 12 h and are not readily excreted back into the surrounding environmental waters even after 48 h (without feeding, although it should be noted that this is not a realistic scenario and that feeding is part of the normal excretion process). Exposure of *D. magna* to gold ENMs (14 nm 0.5–20 mg/L chronic exposure for 14 days) has also been shown to induce physiological effects such as increased moulting, a regulatory mechanism for *D. magna* to shed their exoskeleton as a means to regulate internal metal concentrations [9] implying that ingested metal gold ENMs cause physical effects to *D. magna*. Moulting is an important and necessary part of daphnia life cycle development which is needed not only for growth but also to discard any externally accumulated metals and likely also any surface-adherent ENMs that have bound to the surface of the daphnia [10]. Inability of *D. magna* to successfully moult can lead to their inability to regulate internalized metal concentrations and impedes their ability to survive. The presence of toxicants such as selenium have been known to delay or inhibit moulting completely [11] which can lead to decreased survivorship.

Gold in bulk form is known to be an inert material, although gold ENMs, due to their small size, have an increased reactivity and with the addition of surface charge, especially positive charge which is known to be more toxic than negatively charged gold particles [12], may trigger some biological effects. Gold ENMs with positively charged surface chemistry have been shown to be substantially more toxic towards *D. magna* compared to those with a negative charge, resulting in increased mortality [12]. Toxicity of positively charged ENMs have also been observed with other organisms such as algae and fish where the positive charge potentially has a higher affinity towards the negatively charged phospholipid membrane of which cells are comprised. The addition of surface charge to ENMs is common and therefore investigation of surface charge on the toxicity of ENMs towards organisms such as *D. manga* should be investigated.

Due to the increased surface area provided by ENMs compared to larger materials, they have been shown to generate harmful reactive oxygen species (ROS), which are an excellent indicator of oxidative stress in organisms such as *D. magna* [13]. Oxidative stress is a commonly used cellular molecular response to ENM exposure and can be an indication of damage or response to a foreign object. The charge of surface groups on ENMs can effect toxicity, although even across similar organisms results have not been consistent as to which type of charge causes the greatest effect [14]. For example exposing zebrafish to negatively charged gold ENMs induces genes associated with mitochondrial dysfunction [15] and exposing clams to gold ENMs caused oxidative stress [16].

Currently, there is minimal research investigating the effect of gold ENMs of different shapes, surface charges and charge densities on *D. magna.* Thus, this study aims to: (1) investigate the toxicity of gold ENMs and assess the effects of shape (sphere, short rod, long rod), surface charge (NH_2_ and COOH) and charge to surface area ratio on the toxicity of the ENMs towards *D. magna*; (2) explore the effect of these ENMs on the production of ROS by *D. magna* and (3) examine the influence of ENMs on the ability of *D. magna* to successfully moult as a key step in their development. The results are used to support calls for increasing realism in exposure approaches for ecotoxicity testing of engineered and manufactured ENMs.

## 2. Materials and Methods

### 2.1. Characterization of Functionalized Gold ENMs

Stock suspensions of functionalized gold ENMs were purchased from Nanopartz Inc. (Loveland, CO, USA) and were diluted volumetrically with deionized (DI) water (ultrapure 18.2 MΩ) to create an ENM suspension with a final concentration of 50 µg/mL to be used as working stock solutions which equated to different number concentrations of each type of ENM (see Table S1 for ENM number in each working stock). Experiments were conducted with respect to number concentration to take into account ENM number in each sample, such that comparisons are made on an equivalent particle number or surface area basis. Prior to experiments, ENMs were characterized using a multi-method approach. UV-Vis spectra of the gold ENMs working stocks were obtained using a Jenway 6800 UV-Vis spectrophotometer (Bibby Scientific Ltd., Staffordshire, UK) using disposable cuvettes with a path length of 1 cm. The size of spherical and rod gold ENMs were also obtained using differential centrifugation sedimentation (DCS) which allows for the determination of non-spherical ENMs by adjustment of the non-sphericity factor (CPS Instruments, Analytik Ltd., Cambridge, UK) where size was measured using a 8%–24% sucrose gradient appropriate for gold ENMs with a density of 19.3 g/cm^3^ (working stock; 0.1 mL). The size and morphology of the gold ENMs were also determined using transmission electron microscopy (TEM) performed using a JEOL 1200 (JEOL Ltd., Tokyo, Japan) by pipetting a 200 µL drop of the working stock solution onto holey carbon grid, allowing to air dry overnight before imaging. The hydrodynamic diameter of spherical gold ENMs was also assessed using a Malvern-zetasizer (Malvern Instruments Ltd., Malvern, UK) using dynamic light scattering (DLS) at a concentration of 25 µg/mL diluted with fresh high hardness (HH) Combo medium. All ENMs were characterized in HH Combo medium (except for TEM samples which were dispersed in DI water to minimize drying effects) (see Section 2.3 for details).

### 2.2. Daphnia magna Culturing

Genetically identical *D. magna* (Bham2 strain) were cultured in HH Combo medium (pH 7.6–7.8) [17]. Each culture jar maintained 15 adults in 900 mL of HH Combo medium. Organisms were sustained at 20 °C with constant humidity. Cultures were fed *Cholera vulgaris* (1 mL 1–2 days, 1.5 mL >3 days) and their media was refreshed weekly in order to maintain healthy broods of neonates. Adult *D. magna* were kept for 6 weeks and continued to create healthy broods of neonates every other day. At the end of the 6 weeks, adult *D. magna* were discarded and fresh neonates were grown to replace the prior adults.

### 2.3. Assessing Nanoparticle Effects on Survivorship of D. magna

Twenty *D. magna* neonates were exposed to a range of mass concentrations 0.001–0.05 µg/mL (positively charged ENMs) and 1–50 µg/mL (negatively charged ENMs) and to a range of number concentrations of 2.5 × 10^6^–1.3 × 10^8^ ENMs/mL (positively charged ENMs) and 1.5 × 10^9^–5.5 × 10^10^ ENMs/mL (negatively charged ENMs) diluted with fresh HH Combo medium to a final volume of 5 mL. Exposures were conducted for 24 h as separate experiments (comparisons by mass or by particle number). After the 24 h exposure the percent mortality was determined by counting the number of living organisms. The half maximal effective concentration (EC_50_) of each of the ENMs was then determined.

### 2.4. Assessment of Surface Charge on Gold ENMs

ENMs were placed in a beaker at a concentration of 5 µg/L with a final volume of 50 mL. The number of ENMs present in 50 mL was calculated based on the original concentration of the stock suspension. The total surface area (SA) present in each 50 mL sample was then calculated and the equivalent amount of moles (*n*) present in each sample determined. Potassium chloride (KCl, Sigma Aldrich, Irvine, UK) at a concentration of 0.01 mM was titrated into the sample under mild stirring. The zeta potential of the sample was measured at intervals (<5 min) to monitor the change in the surface charge until 0 mV was reached. The total moles of KCl needed to neutralize the moles of ENMs and total charge per SA was calculated.

### 2.5. Effect of ENMs on ROS Formation

Twenty *D. magna* (Bham2 Strain) neonates were exposed to high (EC_40_) and low (EC_5_) number concentrations of gold ENMs for positively charged ENMs and high (EC_10_) and low (EC_3_) number concentrations for negatively charged ENMs in a final volume of 5 mL for 24 h. These EC values were chosen based on the survivorship of *D. magna* at a range of different number concentrations, in order to give a cross comparison of ROS generation at the same degree of survivorship. At the end of the exposure period living neonates were placed in fresh HH Combo medium for a recovery phase, with recovery assessed at different time points (0, 3, 6, 8 and 12 h) during recovery. At the end of the recovery period, living neonates of each sample were put into a well in a 96-well plate with approximately 250 µL of HH Combo medium. ROS presence was detected using CM-H_2_DCFA (Molecular Probes, ThermoFisher Scientific, Paisley, UK). 50 µg of CM-H_2_DCFA (stored at −20 °C) was thawed for approximately 15 min. Ethanol (100%) was added to the aliquot to create a 1 mM stock solution. A 50 µM working stock was created from the stock by diluting with ethanol and enough working stock was created to add 50 µL to every 250 µL sample containing twenty neonates. Plates were incubated for 40 min at room temperature covered in foil. ROS levels were assessed using the fluorescence function on a plate reader at 485–512 nm excitation/520 nm emission. Exposures were skewed so that all recovery periods ended at the same time so that ROS presence could be assessed at the same time for all experiments.

### 2.6. Effect of Exposure of Gold ENMs on Moulting of D. magna

The influence of positively charged spherical and short rod gold ENMs (5.3 × 10^6^ ENMs/mL; final volume 5 mL) on *D. magna* moulting was assessed by exposing neonates (6 h) for 84 h (length of time it took control neonates to successfully and fully complete a second moulting cycle) to both types of ENMs alongside a control (no ENM exposure). Positively charged NMs were tested due to their higher toxicity compared to negatively charged ENMs. Each jar (*n* = 20) contained only one neonate to assess the process of moulting on an individual organism basis. The removal (shedding) of the exoskeleton was recorded every 6 h until control cultures had completed their second moulting cycle (which occurred at 84 h), and was assessed by visual observation. To promote successful moulting, each individual neonate was fed 20 µL of algae cholera vulgaris (equivalent to 10 µg carbon) 1 h prior to ENM exposure.

### 2.7. Confocal Imaging of Localisation and Retention of ENMs in D. magna Gut

Transmitted light microscopy (Nikon SMZ800, Nikon Instruments, Surrey, UK) was used to visualise the uptake of spherical NH_2_-gold ENMs into *D. magna* neonates. Laser scanning confocal microscopy (LSCM) to assess ENM uptake was conducted using a Zeiss LSM 710 ConfoCor (Zeiss, Jena, Germany) using the 543 nm laser. Gold ENMs were conjugated to the dye Rhodamine B Isothiocyanate (RhB-ITC) where enough dye molecules were used to be able to conjugate to half the NH_2_ surface groups. This value was chosen to have a large proportion of dye molecules conjugated to the gold ENMs but to also reduce the presence of any free dye. Neonates were exposed to RhB-ITC-ENMs for 1 h exposure time and then washed twice with fresh HH Combo medium. Fluorescent images of *D. magna* neonates were taken by placing a single neonate on a 35 mm glass bottom dish and reducing surrounding medium to a minimum to avoid movement using the 10× objective lens to capture images (*n* = 3). Both fluorescent and transmitted light images were recorded and overlaid. Note that we had also tried reflectance confocal imaging of the gold ENMs, but the keratin in the carapacewas also highly reflectant, rendering these measurements unsuccessful as a means to quantify/confirm gold ENM uptake.

### 2.8. Statistical Analysis

Statistical significance was calculated using a standard Student’s *t*-test to compare significance between positive and negatively charged ENMs. Student’s *t*-test was also conducted within each ENM type to with respect to recovery time to determine steady-state levels. *p* values of 0.05, 0.01 and 0.001 with *, ** and *** indicating representative significances in the data.

## 3. Results and Discussion

### 3.1. Characterization of Gold ENMs

The gold ENMs were selected to provide a range of characteristics such as shape, morphology and surface charges. Various types of shaped and charged gold ENMs were used to determine the effect on toxicity towards *D. magna*. A summary of the gold ENM characteristics can be seen in Table 1.

Gold ENMs were characterized using multiple methods to ensure adequate characterization, including DLS, DCS, UV-Vis and TEM.

DLS results provided hydrodynamic diameter of a spherical ENM which is a slightly higher value compared to the core size as ENMs dispersed in medium have a thin layer of solvent that surrounds the surface and hydrodynamic diameter is a measure of ENM diameter and the absorbed surrounding layer. DCS is a useful technique to measure rod shaped ENMs: sedimentation of ENMs follows Stokes’ law, where the dimensions of a rod shaped ENM can be converted to an equivalent spherical diameter following Equation (1):

(1) $$d_{st}=\;d_{c}\sqrt{ln\left(2\beta \right)}$$

where *d_st_* is the equivalent spherical diameter (Stokes’ diamter), *d_c_* is the diameter of the cylinder and β is the aspect ratio (length vs. diameter) of the ENM. According to this equation, equivalent spherical diameter depends directly on the diameter of the rod though only marginally on the length [18], which is why both short (length 60 nm) and long (length 146 nm) rod ENMs appear to have a size value of 33 nm as both rods have the same diameter of 25 nm. Gold ENMs have localized surface plasmon resonance (LSPR) resulting in a strong absorbance band between 500–600 nm which can be seen for spherical, short rod and long rod gold ENMs. Gold ENMs that have a second dimension (rods) have a second peak which is red-shifted to the right with longer diameters resulting in a further red-shift. Characterization of ENMs were completed in fresh HH Combo medium except for TEM images which were prepared in DI water to avoid drying effects from salts present in the media. TEM reflects better the physical dimensions of ENMs providing their dry characteristics and core size, although does not explain the ENMs behavior in dispersion and, depending on sample preparation, may overexpress aggregation, or cause particle dehydration. UV-Vis spectra and TEM images for spherical and rod gold ENMs are provided in Figure S1.

### 3.2. Effect of Gold ENMs on D. magna Survival

**Figure 1:**
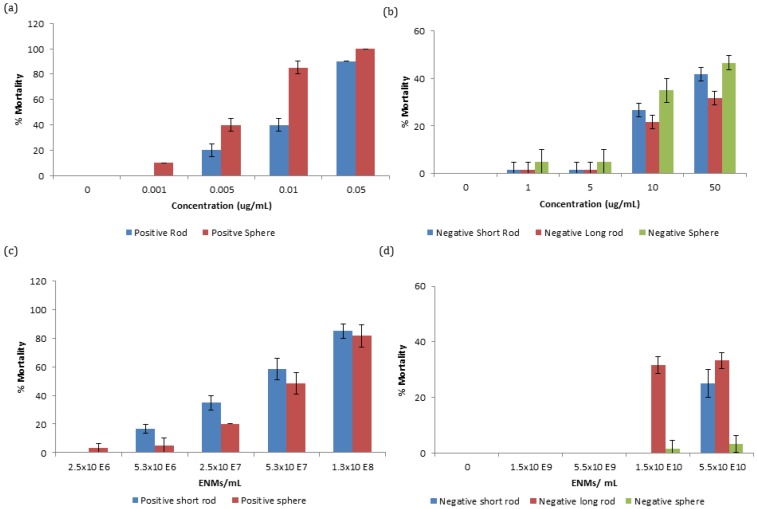
Survival curves of *Daphnia magna* (*D. magna*) neonates exposed to (**a**) positively charged mass concentration; (**b**) negatively charged mass concentration; (**c**) positively charged number concentration; and (**d**) negatively charged number concentration of spherical, short rod, and long rod shaped gold engineered nanomaterials (ENMs). Note: Even at the highest concentration of 50 µg/mL, negatively charged ENMs did not acquire a half maximal effective concentration (EC_50_). Higher number concentrations for negatively charged ENMs can be seen in Figure S2.

Zooplankton were exposed to a range of concentrations of gold ENMs in order to assess survivorship. It was observed that positively charged ENMs, regardless of shape, were orders of magnitude more toxic compared to negatively charged ENMs with respect to mass concentration. The EC_50_ for positively charged spheres and short rods were 6.11 and 18 µg/L respectively. Differences in mortality between positively charged spheres and short rods were statistically significant at concentrations between 0.005 and 0.01 µg/mL at *p* < 0.01 and 0.001, respectively, as determined by students-test. The increased toxicity of positively charged ENMs can be attributed to the positive charge on the NM being attracted to the negative charge on the phospholipid membrane of organisms increasing their interaction [12]. A similar toxicity trend can be observed with negatively charged gold ENMs (Figure 1b) where spheres appear to be most toxic, followed by short rods and then by longer rods although there is no EC_50_ value even at concentrations as high as 50 µg/mL indicating low toxicity of these ENMs. Negatively charged spheres were not significantly different in toxicity compared to short rods, though were statistically significant compared to long rods at 10 and 50 µg/mL at *p* < 0.05 and 0.01, respectively.

Particle number concentration is an important comparison basis to consider for dose-metric issues for ENMs, where mass concentration is not an optimal comparison, especially when considering ENMs of different sizes/shapes. Toxicity of ENMs is not always size dependent and ENM number or surface area exposure have been demonstrated as more optimal choices for NM comparison [19]. Gold ENMs of various number concentrations were exposed to *D. magna* neonates and as expected, regardless of number or mass concentration exposure, positively charged ENMs were still orders of magnitude more toxic than negatively charged ENMs. Interestingly, with respect to number concentration, long rods were found to be most toxic, followed by short rods and finally spheres as seen in Figure 1c,d for both positively and negatively charged ENMs, respectively. Spherical and short rod shaped NMs that were exposed to neonates at the same number concentration have a different amount of surface area exposed to *D. magna* depending on the type of ENM. This can explain why positively charged short rods appear to be most toxic as they have a larger surface area available for interaction (5694 nm^2^) compared to spheres (1963 nm^2^) as can be identified in Table 2. The EC_50_ values for the positively charged ENMs for spheres and short rods with respect to number concentration are 5.69 × 10^7^ ENMs/mL and 4.30 × 10^7^ ENMs/mL respectively. Toxicity of ENMs may also depend on their ability to agglomerate, as *D. magna* preferentially take-up larger entities. It was determined that ENMs remained stable in HH Combo medium for 24 h (Table S2) with no statistical significance in zeta-potential values over time.

Though an increased surface area could explain an increase in toxicity, this would only hold true if the charge per surface area on each of the ENMs types were the same and therefore charge per surface area must also be taken into account to verify the potency of the NM toxicity.

**Figure 2:**
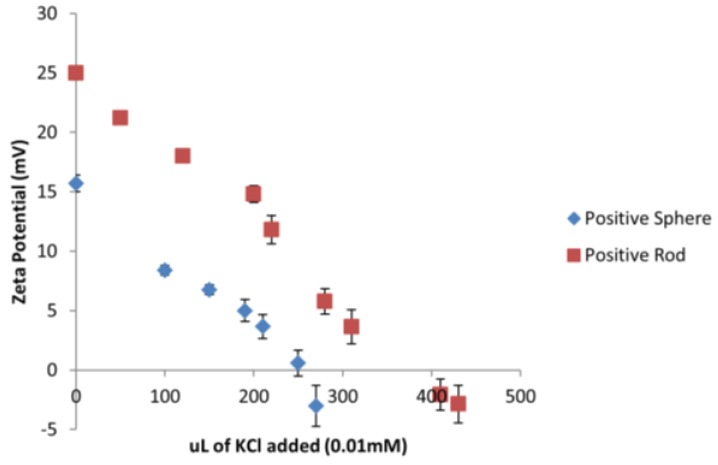
Titration of 0.01 mM KCl to positively charged spherical and short rod gold ENMs and subsequent change in zeta-potential.

Considering that all the tested ENMs are of different sizes and shapes, a different amount of surface area is being exposed to *D. magna* depending on the type of ENM. A series of titrations were conducted using 0.01 mM KCl to neutralize the charge on the gold ENMs in order to determine how many *n* of KCl was needed to neutralize the same amount of *n* of gold ENMs. It was determined that 270 µL and 430 µL of 0.01 mM KCl was needed to neutralize the charges on positively charged spheres and positively charged short rods respectively as can be seen in Figure 2 which corresponds to 2 positive charges/nm^2^ on spheres and 3 positive charges per nm^2^ on short rods (see Supplementary Materials for sample calculation).

Analysis of the charge per surface area can begin to explain the toxicity effects of positively charged ENMs on *D. magna* as short rods have a much higher surface area compared to spheres. Results also indicate that short rods hold a higher number of positive charges per nm^2^ compared to spheres which in turn provides a substantially higher amount of overall positive charge to be available to interact with *D. magna*. Therefore short rods appear to have a dual toxic effect of a larger surface area and an increased charge to surface area ratio compared to the spheres. It was determined for negatively charged long rods, short rods and spheres, that the charge per surface area corresponded to 2 and 3 charges/nm^2^ as seen in Figure S3.

**Figure 3:**
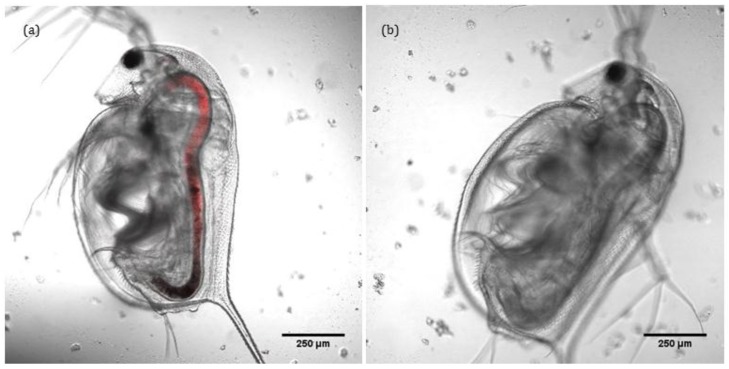
Fluorescent confocal image of *D. magna* neonate retaining NH_2_-gold ENMs conjugated with Rhodamine B Isothiocyanate (RhB-ITC) (**a**) and control (**b**).

Confocal and transmitted light imaging was used to confirm that the intaken ENMs remain localised solely within the gut of *D. magna* and do not translocate into other tissues, as shown in Figure 3a,b. Thus, lumen cells and microvilli along the bush boarder are exposed to ENMs, and observed toxicity could be due to selective interaction of ENMs with intracellular organelles (mitochondria or DNA) of lumen cells and microvilli. Charge density is also a moderator of the degree of toxicity (see Table S3). ENMs do not appear to translocate any further than the gut, illustrating its effectiveness as a barrier. Note that the fluorescence was added via conjugation to ~50% of the –NH_2_ groups on the surface of the positively charged ENMs. Transmitted light images of the same can be found in Figure S4. As gold ENM are inherently reflectant, use of reflectance confocal microscopy as a means to quantify uptake and localisation was also attempted. However, the carapace itself is highly reflectant and as *D. magna* tend to consume the carapace (in the absence of food sources) and the keratin protein that constitutes the carapace is itself highly reflectant, such that control organisms (with no ENMs) were actually more reflectant that the gold ENM-exposed *D. magna*.

### 3.3. Effect of Gold ENMs on ROS Production in D. magna

**Figure 4:**
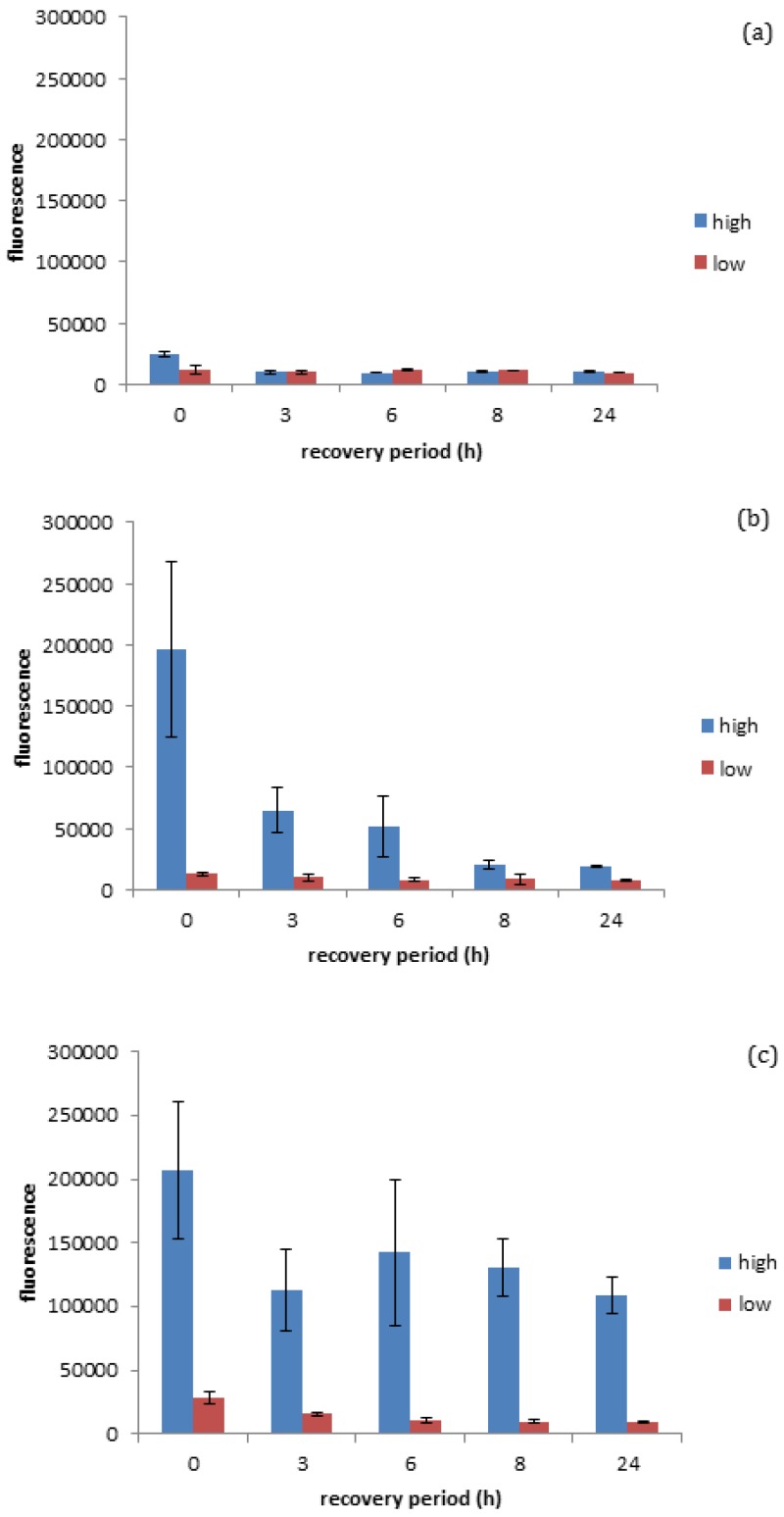
Reactive oxygen species generation and recovery (0–24 h) in response to high and low number concentration exposures of negatively charged spheres (**a**); positively charged spheres (**b**); and positively charged short rods (**c**).

It was determined that gold ENMs regardless of their shape or charge are able to influence *D. magna* survival and therefore an analysis of the toxicity mechanism was performed. It is widely recorded that *D. magna* produce ROS as an indicator of stress [20]. The production of ROS within organisms is an excellent indicator of stress at a biochemical level and can take the form of superoxide anion radical, hydrogen peroxide and hydroxyl radical, each of which are known to be produced by *D. magna* [20]. As seen in Figure 4a, it was quantified that negatively charged gold spheres prompt minimal ROS production in *D. magna* exposed to either high (EC_10_) or low (EC_3_) number concentrations of negatively charged gold ENMs (for EC_10_ of negatively charged spheres see Figure S2), while positively charged spherical gold ENMs prompted significant ROS production in *D. magna* exposed to either high (EC_40_) or low (EC_5_) number concentrations of positively charged gold ENMs. EC values were chosen as appropriate ‘high’ and ‘low’ values based on survivorship results where ENMs of the same charge are comparable. Further explanation of the selection of the ENM number particle concentrations is given in the supplementary information alongside Table S1. This is complementary to the survivorship results in Figure 1 and Figure 2, as negatively charged gold ENMs were shown to be orders of magnitude less toxic compared to positively charged gold ENMs.

As seen in Figure 4b, positively charged spheres prompt a higher degree of ROS generation (determined immediately after the exposure period, so at 0 h recovery) compared to negatively charged spheres at the high exposure dose seen in Figure 4a, although the accumulated ROS is reduced by approximately 65% within the three hours of the recovery period and reaches steady state levels (approximately 10%) after 8 h of recovery (insignificant *t*-test *p* < 0.05). This is in line with survivorship results as high concentrations of positive spherical ENMs can cause a high generation of ROS where coping mechanisms are not able to fully overcome the ROS produced, resulting in increased *D. magna* mortality. ROS generated by positive and negatively charged spheres when exposed to high and low concentrations of ENMs are significantly different at 0 h recovery (significant *t*-test *p* < 0.05 and *p* < 0.001 respectively) and statistically significant at low concentrations at 24 h recovery (*p* < 0.05). Positively charged rods also appear to prompt a high degree of ROS generation, although *D. manga* coping mechanisms appear to be unable to fully recover from the stress in this case, unlike with positively charged spheres. This inability to recover could be attributed to rods having an increased surface area as well as a higher surface area to charge ratio, thus causing them to induce a higher degree of toxicity/ROS activation as seen in Figure 4c. It was observed that Daphnia were unable to successfully remove ROS accumulation even after 24 h recovery, which can be due to decreasing levels of anti-oxidant enzymes and reduced anti-buffering capacity where there is insufficient enzyme available to handle ROS production and therefore a ROS spike occurs at 8 h into recovery. This prompts the de novo synthesis of anti-oxidant enzymes to handle the excess ROS and can be seen as ROS levels start to decrease again. It is interesting as this may be a factor to explain why rods are more toxic compared to spheres at the same number concentration, as *D. magna* are able to effectively remove ROS when exposed to high levels of spheres though are unable to when exposed to rods, which could potentially be due to the ingestion of the ENMs or increased adsorption of ENMs to the carapace leading to a decrease in moulting. Of course, the production or ROS is not the only factor influencing ENM toxicity, whereby both ROS and high toxicity could correlate to cellular stress, whereby damage to the mitochondria could cause a decrease in cellular respiration and ATP depletion. Cellular stress caused by damage to DNA can result in the cells undergoing unregulated necrotic cell death. Upon exposure to cellular stress, homeostasis between net growth and death is altered, whereupon cells may employ a protective cellular response. However, if cellular stress is too high this will result in the activation of cell death pathways [21] with ROS functioning at all stages of this process, either as an initiating event or as a symptom of cellular stress.

### 3.4. Effect of Gold ENMs on Moulting of D. magna

**Figure 5:**
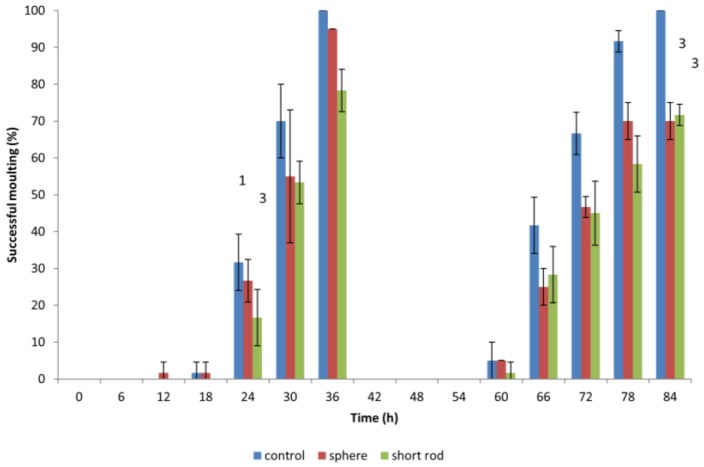
Moulting success of *D. magna* neonates (6 h) exposed to 5.3 × 10^6^ ENMs/mL of spherical and short rod gold ENMs for 84 h. Numbers on top of bars indicate daphnia mortality.

When *D. magna* neonates were previously exposed to gold ENMs, neonates would appear to halt in mid-swim and then return to a regular swimming pattern which prompted the idea if ENMs were adhering to the surface of *D. magna* and if this would have an effect on shedding of the carapace, hindering further development. During the first moulting, which occurs approximately 24 h after birth, *D. magna* shed their exoskeleton, and this is known to be a mechanism for removing metal pollutants also, so we postulated that any adhered gold ENMs would also be shed. Our results indicate that all living *D. magna* neonates that are exposed to gold ENMs at a concentration of 5.3 × 10^6^ ENMs/mL (regardless of shape or charge) successfully complete the first round of moulting. The concept of adsorption of ENMs to organisms has been well established that the presence of environmental stressors induces difficulties in moulting [22]. Biological surface coating leads to ENMs adhering to the filtering apparatus of *D. magna* [10] resulting in an increased chance of uptake by filter feeding. Our results indicate that exposure to gold ENMs plays a major role in the decrease of moulting ability of *D. magna*. The coating of the exoskeleton by gold ENMs increases both the weight along with physical stamina required by each neonate, which consequently increases the energy demand of each neonate. For neonates also struggling with high ROS levels, we postulate that the required energy for moulting is unavailable due to the high demand for energy used in an attempt to remove accumulating ROS for neonates exposed to positively charged spheres or short rods. Our results clearly indicate that an 84 h exposure to 5.3 × 10^6^ ENMs/mL of spherical and rod shaped gold ENMs significantly reduces the success of a second moulting compared to 100% moulting success in the control group (**, *p* < 0.01 for spheres) and (**, *p* < 0.01 for short rods). *D. magna* exposed to positively charged gold rods, moult less, though not significantly, compared to those exposed to spheres at the end of the exposure period (84 h), as seen in Figure 5. The inability of *D. magna* to moult combined with accumulated ROS generation result in *D. magna* immobility and mortality. This is consistent with findings from Dabrunz et al. for nanoscale titanium dioxide ENMs (nTiO_2_), who hypothesized a mechanistic chain of events for nTiO_2_ toxicity in *D. magna* that involves the coating of the organism surface with nTiO_2_ combined with a moulting disruption [10].

## 4. Conclusions

*D. magna* is a crucial indicator species to assess toxicity of ENMs due to its position in fresh water ecosystem food-chains and their ability to ingest matter from the water column. The work presented here is paving the way to understanding and addressing this issue, focussing on the role of ENM shape, surface charge and in determining ENM toxicity to *D. magna*.

Positively charged gold ENMs show to be orders of magnitude more toxic compared to negatively charged gold ENMs due to the presence of the positive charge attracting a negative charge on the phospholipid membrane of the zooplankton. With respect to particle number concentration, which is a more accurate representation of dose-response, larger ENMs show to be more toxic than smaller with order of toxicity ranking with long rods, short rods and finally spheres (for both positive and negative ENMs), due to a larger available surface area to interact with the organism along with a larger surface area to charge ratio being held by positive short rods compared to positive spheres. Negatively charged spheres have a surface area to charge ratio of 3 charges/nm^2^ compared to only 2 on negatively charged short and long rods, causing increased repulsion of spherical ENMs to *D. magna*. A table of ENM types and their charge per surface area can be found in Table S3. Toxicity results coincide with ROS generation and moulting success of *D. magna* indicating that toxicity of positively charged gold ENMs is a surface charge effect and oxidative stress issue coupled with physical effects due to inhibition of moulting (or diversion of the energy required for moulting to amelioration of ROS).

The results we have demonstrated indicate that both shape and charge are important factors in determining ENM toxicity towards organisms, and that these characteristics may be acting on an individual or a combined level to instigate chemical changes, such as oxidative stress or physical changes such as moulting inhibition, which are key parameters in maintaining balance and normal development of *D. magna*. The deposition of various charged and shaped ENMs in environmental waters has the potential to cause important physiological and developmental changes to *D. magna* that may not be picked up with traditional OECD tests which do not take into account the complexity of shape and morphology of ENMs with different shapes or charges. Thus, comparison of ENM on a mass basis alone misses important subtleties resulting from non-equivalent surface areas and charge per surface area. Assessing only mortality also misses important details such as ability to recover from oxidative stress up to specific thresholds, or physical effects such as inhibition of moulting.

## Figures and Tables

**Table 1:** Characterization of spherical and rod shaped gold engineered nanomaterials (ENMs, nanometer (nm)), representative of both positively and negatively charged ENMs which were selected to be identical other than their surface charge. ENMs were characterized using dynamic light scattering (DLS), disc centrifugation sedimentation (DCS), transmission electron microscopy (TEM) and ultraviolet-visible (UV-Vis) absorption spectrometry.

	Sphere	Short Rod	Long Rod
DLS	53 *(PDI 0.195)*	N/A	N/A
DCS	20	33	33
TEM	25	*Diameter* 25	*Diameter* 25
		*Length* 60	*Length* 146
UV-Vis	522	522	520
		660	960

**Table 2:** Calculated dimensional proportions representative of positive and negatively charged spherical and rod shaped gold ENMs.

	Radius (nm)	Length (nm)	Surface Area (nm^2^)	Volume (nm^3^)	SA/V Ratio
Sphere	12.5	-	1963	8181	0.239946
Short Rod	12.5	60	5694	29,452	0.193332
Long Rod	12.5	146	12,448	71,667	0.173692

## Supplementary Materials

The following are available online at http://www.mdpi.com/2079-4991/6/12/222/s1.
