# Supplementary material for: Shape and Charge of Gold Nanomaterials Influence Survivorship, Oxidative Stress and Moulting of Daphnia magna
Source: Nanomaterials (Basel). 2016 Nov 25;6(12):222. doi: 10.3390/nano6120222 (PMC5302705; doi:10.3390/nano6120222)
Supplement: Supplementary file 1 [file nanomaterials-06-00222-s001.pdf]

Supplementary Materials: Shape and Charge of Gold Nanomaterials Influences Survivorship, Oxidative Stress and Moulting of *Daphnia magna*

Fatima Nasser, Adam Davis, Eugenia Valsami-Jones and Iseult Lynch

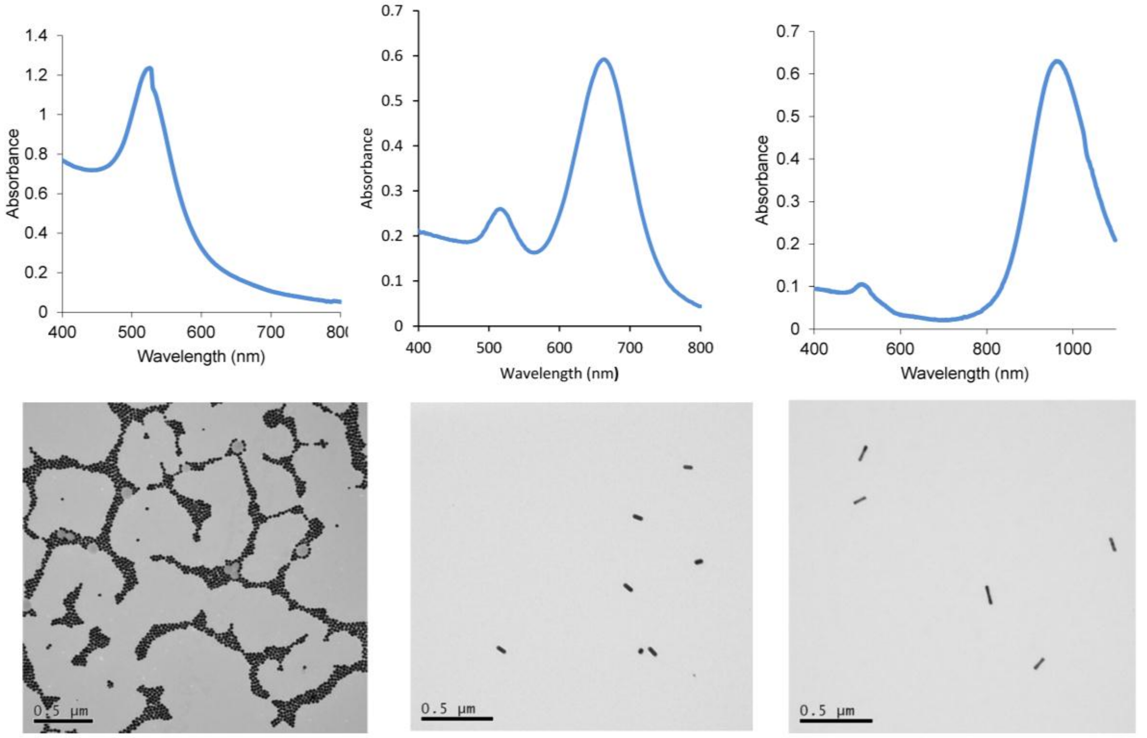

Figure S1. UV-Vis spectra of gold spheres, short rod and long rod gold ENMs (left to right).

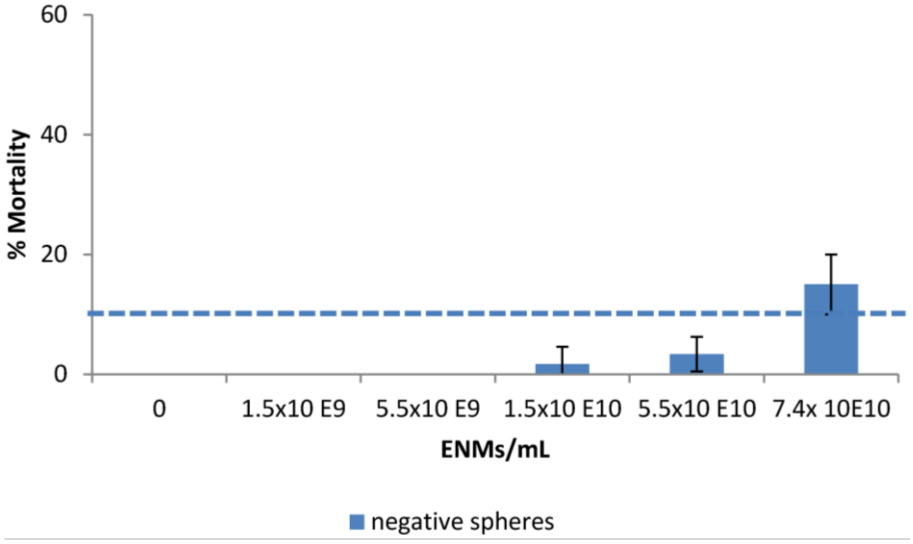

Figure S2. Mortality curve of *D. magna* exposed to various number concentrations of negatively charged ENMs, including values obtained for EC_{10}.

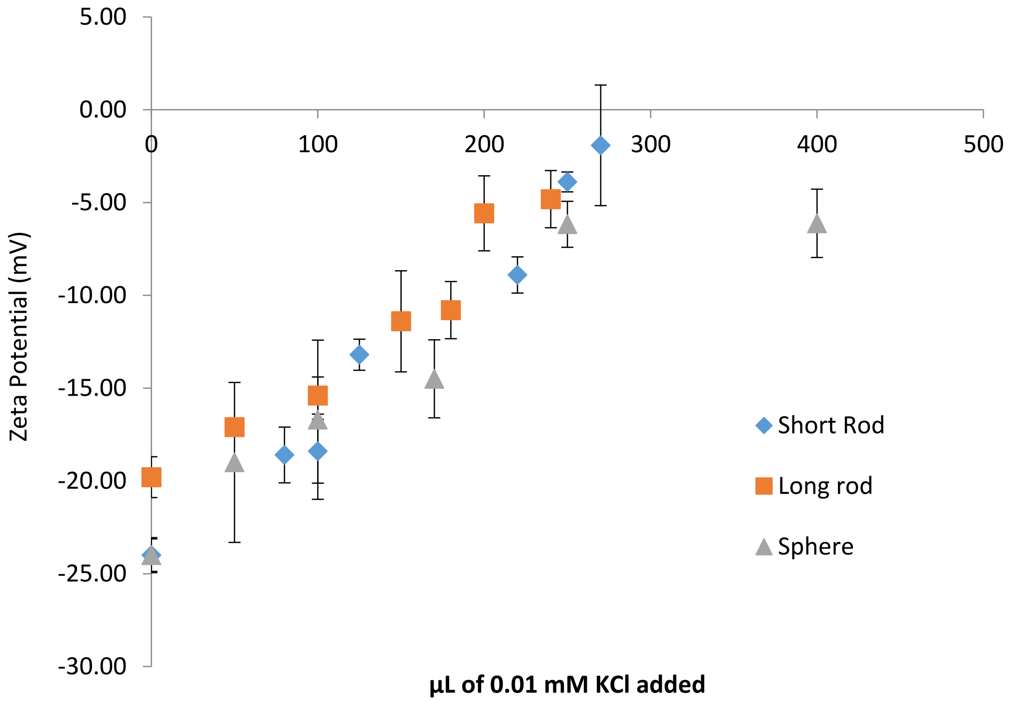

Figure S3. Titration of 0.01 mM KCl into negatively charged spherical and short rod gold ENMs and subsequent change in zeta-potential.

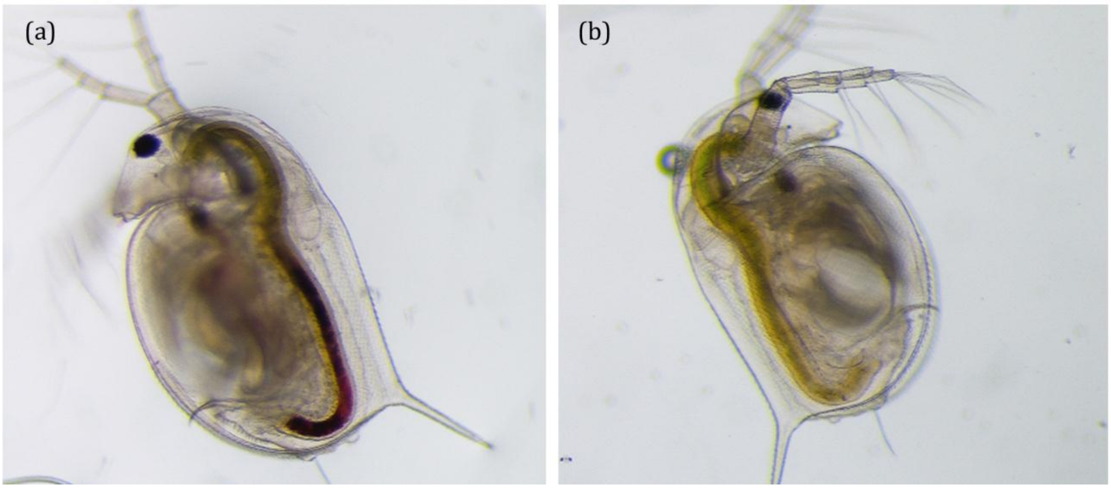

Figure S4. Transmitted light images of *D. magna* exposed to NH_2-gold ENMs conjugated with RhB-ITC (**a**) and control (**b**).

Table S1. Conversions of mass concentration of ENMs to number concentration of working stock (50 µg/mL).

	Number Concentration of Stock (NPs/mL)	Mass Concentration of Stock (mg/mL)	Number Concentration of 50 µg/mL Working Stock (NPs/mL)
Negative Short Rod	6.9×10^{12}	2.5	1.38×10^{11}
Negative Long Rod	2.8×10^{12}	2.5	5.6×10^{10}
Negative Sphere	2.63×10^{13}	2.5	5.26×10^{11}
Positive Short Rod	6.9×10^{12}	2.5	1.38×10^{11}
Positive Sphere	2.67×10^{13}	2.5	5.34×10^{11}

'High' and 'low' particle concentrations were selected based on the mortality results from positively and negatively charged NPs, using a NP number concentration where all NPs of the same

charge would be comparable. Number concentrations compare the same number of NPs in a certain (fixed) volume. Thus, converting from the mass concentrations to number concentrations meant that for the spheres there was an order of magnitude more particles than for rods (see table above) at the same mass. Thus, we had to select the NP number concentrations for comparison across a set of similar charged particles on the basis of the NP with the lowest number concentrations (for the X axis), which limited the Y-axis response. For the negatively charged NPs, which were low toxicity, and thus never reached EC_{40} concentration at the number concentrations evaluated, the EC_{10} concentration was chosen as the "high" concentration and EC_3 as the "low" concentration, as these span the range of effects observed. Note that there is no attempt to compare positive with negative here, but rather effect of shape and charge density within a specific charge.

Table S2. Zeta potential of ENMs dispersed in HH Combo medium at 0 and 24 h.

ENM Type	Zeta Potential at 0 h (mV)	Zeta Potential at 24 h (mV)	Significance
Negative sphere	−27.6	−28.4	None
Negative short rod	−25.0	−25.4	None
Negative long rod	−12.4	−15.5	None
Positive sphere	+20.4	+20.7	None

Table S3. Summary of ENM type and their charge per surface area and ranking of toxicity.

Nanoparticle	Charge Per Surface Area	Ranking of Toxicity
Positive Short Rod	3	1
Positive Sphere	2	2
Negative Long Rod	2	3
Negative Short Rod	2	4
Negative Sphere	3	5

Sample calculation of calculating charge per surface area.
5 μg/L positively charged spheres Au NMs in 50 mL.
5.3×10^7 NMs/mL at 5 μg/L.

$$\frac{5.34 \times 10^7 \times NPs}{\text{mL}} \times 50\text{ mL} = 2.67 \times 10^9 \text{ NMs in 50 mL} \quad (S1)$$

$$2.67 \times 10^9\, NMs \times 1963\text{ nm}^2 = 5.24 \times 10^{12}\text{ nm}^2\, total\ SA \quad (S2)$$

Total charges = ?

$$5\,\frac{\mu\text{g}}{\text{L}} = 5 \times 10^{-6}\,\frac{\text{g}}{\text{L}} \quad (S3)$$

$$n = \frac{m}{M} = \frac{2.5 \times 10^{-7}\text{g}}{197\text{ g/mol}} = 1.296 \times 10^{-9}\text{ mol of gold} \quad (S4)$$

$$c = \frac{n}{V};\ n = CV;\ n = (0.00001\text{ M}) \times (250 \times 10^{-6}\text{ L}) = 2.5 \times 10^{-9}\text{ mol KCl used} \quad (S5)$$

$$\frac{2.5 \times 10^{-9}\text{ mol KCl}}{1.295 \times 10^{-9}\text{ mol Gold}} = 1.98 \approx 2 \quad (S6)$$

$$\frac{Total\ Charge}{Total\ SA} = \frac{(5.24 \times 10^{12}\text{ nm}^2) \times 2}{5.24 \times 10^{12}\text{ nm}^2} = 2\text{ charges/nm}^2 \quad (S7)$$

© 2016 by the authors. Submitted for possible open access publication under the terms and conditions of the Creative Commons Attribution (CC-BY) license (http://creativecommons.org/licenses/by/4.0/).
